# Imaging of COVID-19 pneumonia in children

**DOI:** 10.1259/bjr.20200647

**Published:** 2020-07-30

**Authors:** Figen Palabiyik, Suna Ors Kokurcan, Nevin Hatipoglu, Sinem Oral Cebeci, Ercan Inci

**Affiliations:** 1 Department of Pediatric Radiology, Health Science University, Bakirkoy Dr. Sadi Konuk Training and Research Hospital, Istanbul, Turkey; 2 Department of Radiology, Health Science University, Bakirkoy Dr. Sadi Konuk Training and Research Hospital, Istanbul, Turkey; 3 Department of Pediatric Infection, Health Science University, Bakirkoy Dr. Sadi Konuk Training and Research Hospital, Istanbul, Turkey

## Abstract

**Objective:**

Literature related to the imaging of COVID-19 pneumonia, its findings and contribution to diagnosis and its differences from adults are limited in pediatric patients. The aim of this study was to evaluate chest X-ray and chest CT findings in children with COVID-19 pneumonia.

**Methods:**

Chest X-ray findings of 59 pediatric patients and chest CT findings of 22 patients with a confirmed diagnosis of COVID-19 pneumonia were evaluated retrospectively.

**Results:**

COVID-19 pneumonia was most commonly observed unilaterally and in lower zones of lungs in chest X-ray examinations. Bilateral and multifocal involvement (55%) was the most observed involvement in the CT examinations, as well as, single lesion and single lobe (27%) involvement were also detected. Pure ground-glass appearance was observed in 41%, ground-glass appearance and consolidation together was in 36%. While peripheral and central co-distribution of the lesions (55%) were frequently observed, the involvement of the lower lobes (69%) was significant. In four cases,the coexistence of multiple rounded multifocal ground-glass appearance and rounded consolidation were observed.

**Conclusion:**

COVID-19 pneumonia imaging findings may differ in the pediatric population from adults. In diagnosis, chest X-ray should be preferred, CT should be requested if there is a pathologic finding on radiography that merits further evaluation and if clinically indicated.

**Advances in knowledge:**

Radiological findings of COVID-19 observed in children may differ from adults. Chest X-ray should often be sufficient in children avoiding additional irradiation, chest CT needs only be done in cases of clinical necessity.

## Introduction

The novel coronavirus was first reported in the Wuhan region, on Dec 31, 2019 and transmitted person to person was named as SARS-CoV-2 by the International Committee on Taxonomy of Viruses on Feb 11, 2020.^1^ On the same day, the World Health Organization (WHO) declared the disease caused by this novel virus as COVID-19.^2^ Upon spreading all over the world, on Mar 11, 2020, COVID-19 disease was accepted as a pandemic disease by WHO.

COVID-19 pneumonia is highly contagious and widespread in the adult population and often presents with significant clinical finding. Whereas in pediatric patients, the incidence appears to be low and it presents mostly in an asymptomatic condition or with mild clinical symptoms. The incidence in children has been reported as 1.7–2.4% in the literature.^3,4^ Chest CT is an important diagnostic method in the diagnosis of COVID-19 pneumonia in adult patients. However, to protect from radiation, imaging is often not required in pediatric cases if there is no clinical finding. Chest X-ray is the first preferred imaging method in pediatric patients who are clinically considered to have COVID-19 pneumonia. Chest CT can be performed in patients with chest X-ray findings. The number of cases and literature reporting the imaging findings observed in COVID-19 pneumonia in children worldwide is limited due to the low number of pediatric cases and its good clinical course.^5^ In these limited studies, it is stated that chest CT imaging findings are similar to adults. However, the findings on chest X-ray, typically preferred in pediatric patients, have not been widely reported.^6^

In this retrospective study, we evaluated the imaging findings observed in chest X-ray and chest CT in children diagnosed with COVID-19 pneumonia.

## Methods and materials

### Data collection

In this retrospective study, we evaluated chest X-ray of 177 children and chest CT of 74 children with a pre-diagnosis of COVID-19 pneumonia who were admitted to pediatric outpatient clinics of our COVID-19 pandemic hospital between Mar 11 and Apr 20, 2020. Permission from the Ethics Committee of Bakirkoy Dr. Sadi Konuk Training and Research Hospital was obtained (2020/153) and consent forms were taken from the parents of cases. The patients were between 0 and 17 years of age. Real-time fluorescence polymerase chain (RT-PCR) test and imaging (chest X-ray or chest CT) were performed at the time of application. The demographic features, clinical features and radiological images of the cases with the diagnosis of COVID-19 infection confirmed by RT-PCR was obtained from the electronic medical record system, and chest X-ray and chest CT images were evaluated using Picture Archiving and Communication Systems (PACS). Radiological evaluation was performed by a pediatric radiologist who has 10 years of experience in pediatric chest imaging. All cases confirmed as Covid-19 by RT-PCR were classified clinically as mild, moderate, severe and critical.^5,7^

### Imaging techniques

PA (posteroanterior) or AP (anteroposterior) chest X-rays were performed on digital X-ray machines that perform automatic exposure at radiation doses suitable for children (Samsung, XGEO GC80, South Korea- SITEC, DIGIRAD-FP/M, South Korea).

All chest CT examinations were performed on 128 and 256 multislice CT devices (Siemens Somatom Sensation, Siemens, Erlangen, Germany) without contrast medium. Chest CT parameters were 120 kVp, 140 mAs, 5 mm collimation, 1.35:1 pitch, a pulmonary kernel (B70f) and a mediastinal kernel (B30f), reconstruction slice thickness of 1.00 mm and high spatial resolution algorithm. The procedure was performed in younger children while calm, in older children while holding breath.

### Chest X-ray analysis

The parameters used in the evaluation of chest X-ray were: a) whether it is normal or pathological, b) the affected lung side (unilateral–bilateral), c) the number of lesions (single–multiple), affected lung zone, d) other findings (pleural effusion, lymphadenopathy).

### Chest CT analysis

The basic data used in a detailed analysis and evaluation of the imaging appearance of each of the identified lesions were: a) the affected lung side (unilateral–bilateral), b) the number of lesions (single–multiple) b) the affected lung lobe and segments, c) the affected lung field (peripheral–central–mix), d) lesion density e) lesion size, f) additional findings (prominent interstitium, prominent vascular structures within the lesion, halo sign, inverted halo sign, pleural effusion, lymphadenopathy). The distribution of affected lung was classified as the upper lobe of the right lung, middle lobe of the right lung, lower lobe of the right lung, upper lobe of the left lung, lingular segment of the left lung, lower lobe of the left lung. Lung field distribution was evaluated as peripheral (1/3 outer region of the lung), central (inner 2/3 of the lung) and mixed (peripheral and central involvement together). Density of the lesions were evaluated as pure ground-glass opacity (GGO), a mixture of GGO-consolidation, and pure consolidation. In addition, the severity were classified as mild, moderate and severe, according to the findings observed in CT examination. For classification, both lungs were divided into three parts as upper, middle and lower. Scoring was made according to the volumetric involvement in each field. If the involvement was 0–25%, it was scored as one point, for 25–50% two points, 50–75% 3 points and 75–100% four points. Scores were summed up as 1–6 points, 7–11 points and 12 and above, and they were evaluated as mild, moderate and severe involvement, respectively.^8^

### Statistical analysis

All statistical analysis was performed using Statistical Package for Social Sciences software v. 24.0.

## Results

### Patients information

59 patients with Covid-19 pneumonia diagnosis confirmed by RT-PCR test and with clinical findings had chest X-ray imaging. The mean age of these patients (34 boys, 25 girls) was 9 years (range 54 days–15 years of age). The ages of 22 patients (15 boys, 7 girls) who underwent chest CT imaging ranged from 6 months to 17 years, with an average age of 12.3 years. 16 (27%) of the patients with chest X-ray also had chest CT examination. six patients got only chest CT imaging. There were approximately 3.2 ± 3.6 days between the dates of first symptom seen and imaging (0–10 days).

In the clinical classification of these patients, 52 patients were evaluated as having mild disease, while 10 patients had moderate and 3 patients had severe disease. All of the patients with mild disease were followed- up with home isolation, all the patients with moderate disease were hospitalized in the general ward and three patient with severe disease were admitted into the intensive care unit (ICU). Only, one of the patients who has the diagnosis of cerebral palsy with severe disease needed to be intubated. One of the patients who was admitted to the intensive care unit had a diagnosis of chronic renal failure.

Control imaging was not performed in any of the clinically mild and moderate cases. The patients in the intensive care unit were followed up with a chest X-ray.

### Chest X-ray evaluation

**Figure 1.**
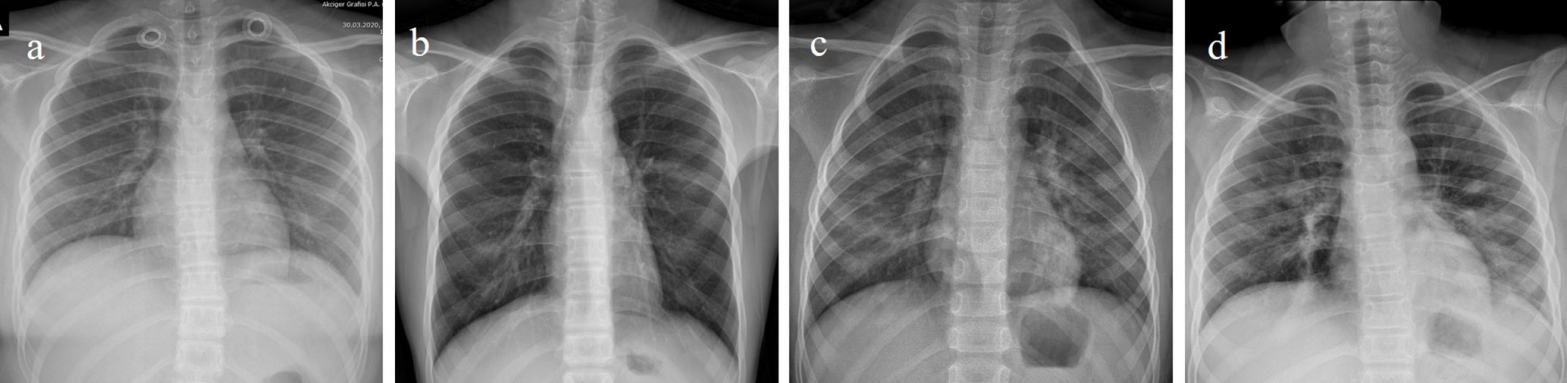
Chest X-ray imaging of COVID-19 pneumonia in children.(a) Female, 11 years old. Chest X-ray showed localized density is observedin the left lung lower zone. (b) Male, 13 years old. Chest X-ray showed scattered densities in bilateral lung lower zones. (c) Male, 5 years old. Chest X-ray showed scattered densities in bilateral lung middle and lower zones. (d) Male, 9 years old. Chest X-ray showed scattered round multiple densities in bilateral lung middle and lower zones.

Lung findings were detected in 27 (46%) of the patients who underwent chest X-ray imaging.Table 1 It was unilateral in 15 (55%) and bilateral in 12 (45%) patients. Of the lesions, 13 were single (48%) and 14 were multiple (52%). No significant change was observed between right and left lung involvement. The lower zones were the most commonly affected areas. The right lower zone was involved in 15 patients (56%) and the left lower zone was affected in 16 patients (59%). The right middle zone was affected in nine cases (33%) and left middle zone in seven cases (26%). Localized increase in density was observed in 27 patients while a diffuse increase in density was observed in cases who were admitted to the intensive care unit. Pleural effusion and lymphadenopathy were not identified.(Figure 1)

**Table 1.** Chest X-ray findings with COVID-19 pneumonia in children

Findings	Number of patients (%)
**Pulmonary lesions**	
Normal	32 (54%)
Patological	27 (46%)
**Affected lung side**	
Unilateral	15 (55%)
Bilateral	12 (45%)
**Number of lesions**	
Single	13 (48%)
Multiple	14 (52%)
**Affected lung zone**	
Right middle zone	9 (33%)
Left middle zone	7 (26%)
Right lower zone	15 (56%)
Left lower zone	16 (59%)
White lung	1 (2%)

### Chest CT evaluation

In chest CT examination, no pathology was observed in three patients (14%). Unilateral lung involvement was observed in seven patients (32%), while six (27%) of those had single lobe involvement. Bilateral lung involvement and multiple lobe involvement were detected in 12 (55%) cases. In all six patients with single lobe involvement, one lesion each was observed. One patient had a “white lung” appearance accompanied by interlobular septal thickening, bilaterally.

In the cases, 9 lesions (8%) in the right upper lobe, 11 lesions (9%) in the right middle lobe, 31 lesions (26%) in the right lower lobe, 8 lesions (7%) in the left upper lobe, 9 lesions (8%) in the left lingular segment and 50 lesions (42%) in the left lower lobe were observed. In 7 (39%) of the cases, the lesions were located only peripherally, while 12 (55%) of them had the lesions in mixed locations.

**Figure 2.**
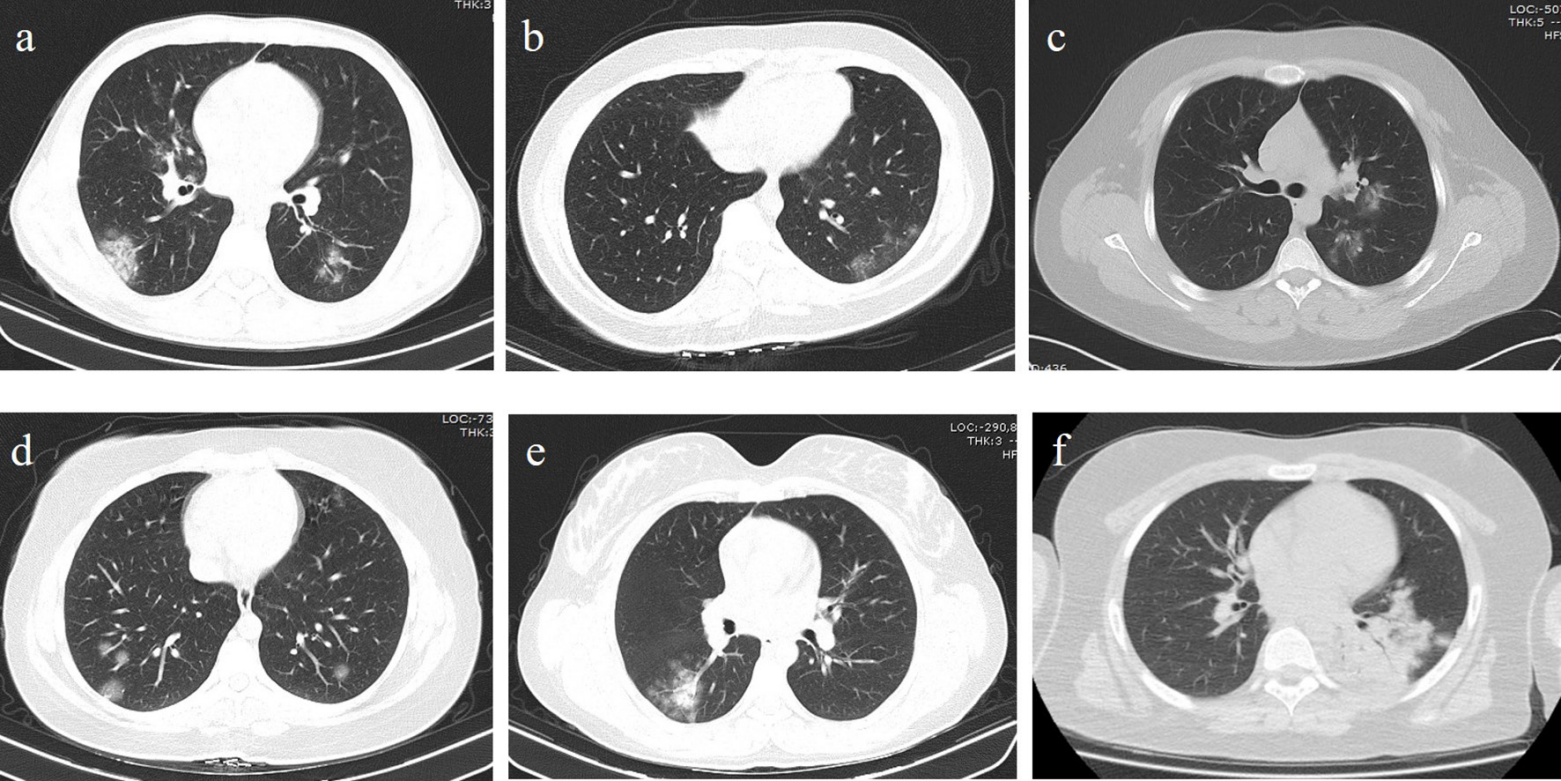
Chest CT imaging of COVID-19 pneumonia in children. (a) Male, 15 years old. Chest CT showed multiple GGO and thickening of interlobular septa in the inferior lobe of the bilateral lung, located peripherally. (b) Female, 17 years old. Chest CT showed single GGO in the inferior lobe of the left lung located peripherally. (c) Male, 15 years old. Chest CT showed single GGO in the superior lobe of the left lung located centrally. (d) Female, 16 years old.Chest CT showed multiple scattered round GGO in the inferior lobe of the bilateral lung.(f) Female, 13 years old. Chest CT showed multiple GGO and consolidation in the right lower lobe. (e) Male, 13 years old.Chest CT showed diffused consolidation in the left lower lobe. GGO, grond glass opacity.

**Figure 3.**
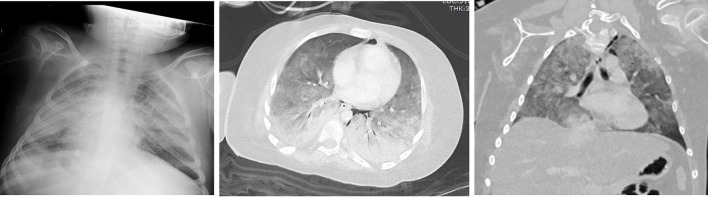
Male, 8 years old intubated. Chest X-ray and CT showed diffuse consolidation.

**Figure 4.**
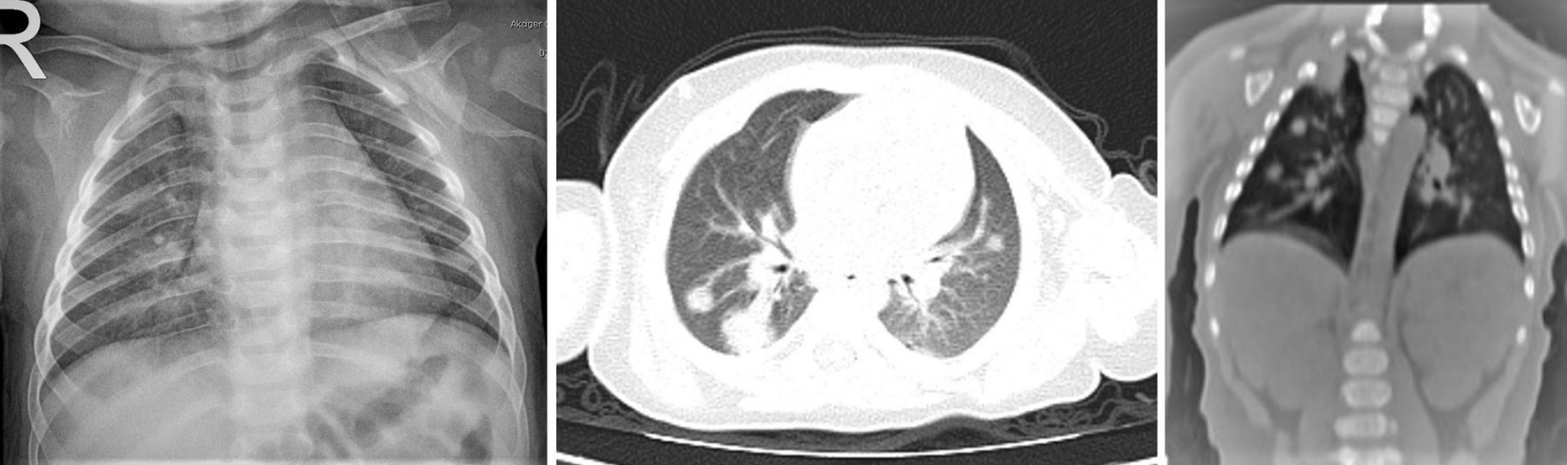
Male, 6- month-old. Chest X-ray and CT showed multiple round-shaped GGO with round multiple consolidation areas.

When the density characteristics of the lesions were evaluated, the pure ground-glass appearance was observed in nine cases (41%), there was pure consolidation in one case (5%) and ground-glass appearance and consolidation were observed together in eight cases (36%).Figure 2 In a case with a severe course (5%), “white lung” appearance was detected.Figure 3 Halo sign in five patients (23%), interstitial thickening in eight cases (36%), prominent vascularity within lesions in eight cases (36%), and atelectasis in one case (5%) were observed. We detected four patients (18%) in which multiple round-shaped ground-glass appearance were observed with round multiple consolidation areas.Figure 4 No pleural effusion or lymphadenopathy was detected.

When severity scoring of cases with pathology detected in chest CT imaging, nine cases(47%) were evaluated as mild, nine cases (47%) were moderate and one case (6%) was severe.There was no correlation between COVID-19 pneumonia clinical and radiological scores.Table 2

**Table 2.** Chest CT findings with COVID-19 pneumonia in children

Findings	Number of patients (%)
**Pulmonary lesions**	
Normal	3 (14%)
Patological	19 (86%)
**Affected lung side**	
Unilateral	7 (32%)
Bilateral	12 (55%)
**Number of lesions**	
Single	6 (27%)
Multiple	12 (55%)
Diffuse	1 (5%)
**Affected lung field**	
Peripherally	7 (32%)
Mixture	12 (55%)
**Lesion density**	
GGO	9 (41%)
GGO ve consolidation	8 (36%)
Consolidation	1 (5%)
White lung	1 (5%)
**Affected lung lobe**	
Right upper lobe	9 (8%)
Right middle lobe	11 (9%)
Right lower lobe	31 (26%)
Left upper lobe	8 (7%)
Lingular segment	9 (8%)
Left lower lobe	50 (55%)
**Scoring of radiological findings**	
Mild	9 (41%)
Moderate	9 (41%)
Severe	1 (5%)

## Discussion

Typical findings in chest CT imaging have been reported in adult COVID-19 pneumonia. The most common findings are multifocal peripherally located ground-glass appearance starting from the lower lobes, accompanied with thickening in the interlobular septa, prominent vascular structures, halo and inverted halo signs. In advanced cases, crazy paving appearance and fragmented consolidation are observed. It is thought that the ground-glass appearance observed especially in chest CT is due to alveolar oedema, exudation and bleeding secondary to inflammation.^8–10^ In the diagnosis of COVID-19, Ai et al identified that RT-PCR positivity was 30–60% in adults, while the sensitivity of thorax CT was 97%, specificity was 25% and accuracy was 68%. They also reported that in the early period, thorax CT findings appeared to correlate with clinical findings without RT-PCR positivity.^11^ Hyungjin et al found that sensitivity and specificity were 94 and 37%. They also reported that the positive predictive value of RT-PCR was more than 10 times higher than of CT imaging.^12^ All these studies in adults show that imaging is very important in the diagnosis of COVID-19 pneumonia.

The number of cases and literature related to the imaging of COVID-19 pneumonia, its findings and contribution to diagnosis, its correlation with RT-PCR and its differences from adults are limited in pediatric patients.There have been few studies, with small numbers, evaluating the findings related to chest X-rays.^6^ However, COVID-19 pneumonia is clinically mild in children, therefore mostly chest X-ray is performed in pediatric patients. Chest CT is performed if there is a pathologic finding on radiography that merits further evaluation and if clinically indicated.Imaging findings have been reported to be different from adults and may be atypical.^13–17^

In pediatric cases, it is essential to use the least radiation-containing test possible for diagnosis in accordance with the principles of radiation protection and ALARA. Chest X-ray imaging is mostly preferred for the diagnosis of pneumonia in children. However, there are few studies on the use of chest X-ray and its findings in COVID-19 pneumonia in the literature. In our chest X-ray examinations, we have observed abnormal findings in 46% of cases diagnosed with COVID-19 pneumonia. All of the cases with a mild illness were treated on an outpatient basis without the need for additional imaging. The most common finding in chest X-ray was a unilateral increase in density. The lower zones were the most affected area. In 13 of 27 patients (48%) with pathology in the chest X-ray, chest CT was required. Though the pathology was observed in the remaining cases, chest CT was not performed since there was no clinical requirement. Xia et al^18^ performed chest CT examination in 20 children with COVID-19 pneumonia without chest X-ray images. They report that due to RT-PCR’s low application and positivity rate in pediatric population, detection and characterization of lesions on the chest X-ray were low in mild cases, and the diagnosis could be missed. Therefore, they emphasized that chest CT will provide early initiation of the treatment as well as early isolation of these cases with typical findings even in RT-PCR negativity. Although the consensus is that without accompanied consolidation in chest X-ray, the ground-glass appearance may be difficult to detect and can be overlooked. We think that, especially in pediatric cases, if a child is well then omitting a CT and missing a few areas of ground-glass change is not of any clinical significance. It is important to protect especially pediatric cases from unnecessary radiation.If there is a clinical requirement along with lung findings, chest CT examination will be appropriate.

There are limited number of studies about thorax CT findings of COVID-19 pneumonia in pediatric patients. Li^19^ and Lui^20^ et al reported in their chest CT studies with five children each that scattered ground-glass appearance was the most common finding, similar to in adults. Chang et al.^21^ in their meta-analysis reported that ground-glass appearance was the most common finding in children, followed by patchy consolidation.

Xia et al^18^ reported bilateral involvement as 50%, ground-glass appearance as 60% and a combination of ground glass appearance and consolidation as 50% in their study on 20 patients. Li et al^19^ in their study reported frequent bilateral and multifocal involvement in chest CT of 22 pediatric patients and stated that unlike in adults, pure consolidation and combination of ground-glass appearance and consolidation were observed more frequently, interlobular septal thickening and crazy paving appearance were less and affected fewer lobes. Duan et al^22^ stated that chest CT findings may be atypical and low in specificity in pediatric cases. They reported that ground-glass appearance might be more localized and lower in density and interlobular septal thickening might be less in children. However, as their severity increased, they reported that ground-glass appearance became multiple consolidations,enlarged and increased in density.

Similar to other studies, pneumonia was most frequently bilateral and multifocal in our series. However, single lesion and single lobe involvement were observed in 27% of the cases. Pure ground-glass appearance and combination of consolidation and ground-glass appearance were approximately equal. In the evaluation of the distribution of lesions that had not been evaluated in previous studies, a combination of peripheral and central configuration was observed more frequently in pediatric patients, in contrast to adults. In addition, 69% of the lesions observed in the cases were in the lower lobes, and the most common involvement was in the left lower lobe. Although the number of cases was low, remarkable multiple multifocal round ground-glass and round pneumonia-like consolidation areas were observed together, in four cases. In cases, similar to adults, interlobular septal thickening, prominent vascular structures within the lesion and halo sign were seen. White lung appearance was detected in an intubated patient in ICU as in the study of Sun et al.^23^ Also, for the first time, we classified the CT findings according to their severity in children as Chung et al did in the adults.^8^ There was no correlation between COVID-19 pneumonia clinical and radiological scores.

Li et al^24^ reported that the reason for less lobe involvement and less prevalence in children may be due to mild inflammatory response due to low immune response. They considered that the reason for accompanying consolidation to ground-glass appearance may be due to progression of pneumonia related to the low rate of the immune response. However, the reason may be due to the inadequate development of the Kohn and Lambert channels between the alveoli due to immaturity, just as in round pneumonia. In four cases followed in our cases, we detected the findings of multiple round ground-glass appearances and round consolidation findings.

Duan et al^22^ reported that although RT-PCR is positive, imaging may not show any findings. In our chest radiography, more than half of our cases (54%) and three chest CT imaging were normal. However, as it is known from adult cases, it is known that the imaging might be normal at the beginning of the disease. Coinfection was reported as 40% by Xia et al.^18^ However, none of our cases supported findings of coinfection. Xia et al^18^ and Duan et al^22^ reported that pleural effusion may be seen in severe cases. Pleural effusion and lymphadenopathy were not observed in any of our cases.

The clinical studies about the use of lung ultrasound findings in pediatric patients with COVID-19 pneumonia are very limited.^25,26^ Musolino et al reported thatlung ultrasound can be used to support diagnosis in the evaluation of suspicious cases or for follow- up patient.^25^ The most common lung ultrasonography findings were confluent B lines, thick irregular pleural lines and subpleural consolidations, as in adult studies..^27–29^ In our study, we did not use ultrasound to evaluate patients.

Our study has some limitations. Firstly, the number of cases was low in both chest X-ray and chest CT examination. In addition, cases with negative RT-PCR but who were likely covid positive due to symptoms and close family contact plus abnormal chest X-ray or CT findings were excluded from the study. It is known some who test negative with RT-PCR can be false negative cases with abnormal chest CT findings.^11–13^
